# Vitamin D supplementation among postoperative atrial fibrillation in patients undergoing coronary artery bypass grafting: a systematic review and meta-analysis

**DOI:** 10.1097/MS9.0000000000003426

**Published:** 2025-05-30

**Authors:** Taimoor Ashraf, Muhammad Abdul Moiz, Ganesh Kumar, Akshay Kumar, Vishal Kumar, Vivek Anand, Fnu Sunita, Naresh Kumar Ladhwani, Tooba Hussain, Shah Dev, Muskan Turesh, Sadia Habib Bhutto, Umer Ejaz, Sayed Jawad

**Affiliations:** a Department of Medicine, Nishtar Medical University, Multan, Pakistan; b Department of Medicine, Services Institute of Medical Sciences, Lahore, Pakistan; c Department of Medicine, Shaheed Mohtarma Benazir Bhutto Medical College, Lyari, Karachi, Pakistan; d Department of Medicine, Bahria University Medical and Dental College, Karachi, Pakistan; e Department of Medicine, Jinnah Sindh Medical University, Karachi, Pakistan; f Department of Medicine, Ghulam Muhammad Mahar Medical College, Sukkur, Pakistan; g Addenbrooke’s Hospital, Cambridge University Hospitals NHS Foundation Trust, Cambridge, UK; h Department of Medicine, Dow University of Health Sciences, Karachi, Pakistan; i Department of Medicine, Rawalpindi Medical University, Rawalpindi, Pakistan; j Department of Medicine, Kabul University of Medical Sciences, Kabul, Afghanistan

**Keywords:** arrhythmia, coronary artery bypass grafting, postoperative atrial fibrillation, vitamin D

## Abstract

**Aims:**

To determine whether vitamin D supplementation reduces the incidence of postoperative atrial fibrillation (POAF) in patients undergoing coronary artery bypass grafting (CABG).

**Methods and Results:**

PubMed and Cochrane Central Register of Controlled Trials were systematically searched from inception through May 2023 for randomized controlled trials (RCTs) assessing the effectiveness of Vitamin D in preventing atrial fibrillation among postoperative patients after CABG. The primary outcome extracted was the incidence of atrial fibrillation after Vitamin D in CABG patients. Secondary outcome included the length of hospital stay. Data were pooled using a random-effect model. A total of 4 RCTs, including 694 patients, were included in the final analysis. The results showed that Vitamin D supplementation significantly reduced the incidence of POAF in CABG patients (RR: 0.55; 95% CI: 0.40 to 0.76; *P* = 0.0003; *I*^2^ = 1%). However, administration of Vitamin D did not lead to significant reduction in the length of hospital stay (WMD: − 0.14; 95% CI: − 0.82 to 0.53; *P* = 0.68; *I*^2^ = 34%).

**Conclusion:**

Our updated pooled analysis concludes that Vitamin D reduces the incidence of POAF in CABG patients. Future large-scale studies should focus on more diverse patient populations and explore a broader range of outcomes to better understand the full impact of Vitamin D supplementation in this context.

## Introduction

Postoperative atrial fibrillation (AF) is a new onset of irregular heart rhythm that typically occurs after surgery, with the highest incidence occurring between the second and fourth days postoperatively. This complication is particularly common following coronary artery bypass grafting (CABG), affecting 20% to 40% of the patients^[1]^. Individuals who develop postoperative AF are at an eightfold increased risk of progressing to long-term AF and face twice the risk of cardiovascular mortality. Additionally, these patients are at a higher risk of stroke, experience prolonged hospital stays, and contribute to increased health care costs^[2-4]^. Several factors, both pre-existing and surgery-related, contribute to the onset of postoperative AF. Surgery-related mechanisms include inflammation, oxidative stress, and adrenergic stimulation. Despite preventive strategies such as beta blockers and amiodarone that target these mechanisms, the incidence of postoperative AF remains high^[1,5]^.

**HIGHLIGHTS**

- Early coronary angiography (CAG) in NSTE myocardial infarction improves outcomes, including lower mortality, reduced recurrence, and less frequent revascularization.
- It enhances long-term survival and neurological recovery, reflected in better Cerebral Performance Category (CPC) scores at discharge.
- Observational studies show more benefits than delayed or no intervention, though randomized trials offer mixed results.

Vitamin D has been recognized for its protective cardiovascular effects, beyond its well-known role in calcium metabolism. It has been shown to lower renin levels, improve glycemic control, and reduce unfavorable cardiac remodeling^[6]^. Vitamin D also exhibits anti-inflammatory properties by increasing the production of the anti-inflammatory cytokine IL-10 and inhibiting the rise of the pro-inflammatory mediator TNF-alpha^[7]^. Several studies have suggested a correlation between low vitamin D levels and an increased risk of postoperative AF^[8,9]^. However, conflicting results from other studies have led to heterogeneity in existing data^[10,11]^. A previous meta-analysis hinted at a potential association between vitamin D supplementation and a reduction in postoperative AF, but its findings were limited by a small sample size^[12]^. Hence, in this updated systematic review and meta-analysis, we investigated the impact of oral vitamin D supplementation preoperatively on the development of postoperative AF.

## Methods

This meta-analysis was performed according to the preferred reporting items for systematic review and meta-analyses PRISMA and AMSTAR guidelines^[13,14]^. This study was registered in the National Institute for Health Research (NIHR) International Prospective Register of Systematic Reviews (PROSPERO) (Identification No. CRD42024564045)^[15]^.

### Literature search

An electronic search was conducted on MEDLINE and Cochrane CENTRAL databases from inception to May 2023, without any language restrictions. Detailed search strategies for the database are provided in the Table S1. http://links.lww.com/MS9/A838. In addition, we manually screened the reference list of retrieved trials, previous meta-analysis, and review articles to identify any relevant studies. The articles retrieved from the systematic search were exported to EndNote Reference Library software where duplicates were screened for and removed. The remaining articles were carefully assessed by two independent reviewers (P.K.L. and F.N.), and only those trials that met the previously defined criteria were selected. All trials were initially short-listed on the basis of title and abstract, after which the full article was reviewed to affirm relevance. A third investigator (S.A.) was consulted to resolve any discrepancies. Studies were included if they met the following criteria: (i) They were randomized controlled trials; (ii) They involved patients who underwent CABG surgery along with vitamin D deficiency determined before surgery (Vit D level < 21 ng/ml) or insufficiency (Vit D level 21–29 ng/ml); (iii) Intervention group patients received vitamin D supplementation along with the standard care in comparison to the control group who had only standard care; (iv) Patients were monitored for postoperative atrial fibrillation (POAF) until discharge. The definitions for POAF considered for individual studies are mentioned in Supplementary Table 2. http://links.lww.com/MS9/A838. Case-control, cohort, cross-sectional studies, meta-analysis, systematic reviews, follow-up, ongoing studies, and those written in other languages apart from English were excluded.

### Outcome extraction and risk of bias assessment

From the finalized trials, the following outcomes were extracted: baseline characteristics displayed in Table 1, incidence of postoperative atrial fibrillation, and length of hospital stay. The revised Cochrane Risk of Bias Tool (RoB 2.0) was used to assess the quality of published trials^[16]^.

**Table 1:** Baseline characteristics of the included studies

First author	CeritL *et al*		Kara H *et al*		Talasaz AH *et al*		Alirezaei T *et al*	
Vit D group design	RCT		RCT		RCT		RCT	
Location	CYPRUS		Turkey		Iran		Tehran	
Publication year	2018		2020		2022		2024	
Intervention	Vit. D	Control	Vit.D	Control	Vit.D	Control	Vit.D	Control
No. of participants	68	68	58	58	93	103	123	123
Mean age	63.8 ± 9.3	62.7 ± 8.9	64.94 ± 10.52	65.21 ± 9.98	59.29 (10.10)	62.22(9.12)	57.44 ± 6.46	58.84 ± 5.69
Male Gender	37	36	16	13	70	74	102	90
Hospital stay (days)	8.1 ± 2.3	7.9 ± 2.1	7.14 ± 1.97	7.81 ± 2.25	7.4	9.58	19.70 ± 7.43	19.27 ± 6.64
BMI	-	-	26.74 ± 3.96	26.62 ± 2.71	28.04	27.63	26.36 ± 2.83	26.96 ± 2.30
Diabetes	25	24	17	22	43	60	58	55
Hypertension	42	43	36	34	47	58	63	64
Smoking history	16	18	-	-	25	29	42	37
COPD	19	18	-	-	3	4	-	-
Hyperlipidemia	16	18	-	-	49	54	57	60
Statin therapy	62	63	27	31	81	91	113	109
Diuretic therapy	15	14	-	-	-	-	60	45
Aspirin therapy	65	63	-	-	-	-	114	112
ACE-I/ARB therapy	46	47	23	17	46	61	99	92
Beta blocker	58	60	21	26	75	89	109	101
Cross-clamping time (min)	74 (71.0-78.0)	75 (72-80)	75.67 ± 23.57	71.21 ± 29.52	-	-	62.07 ± 13.69	59.02 ± 12.15
Pre-op Vit D levels	-	-	-	-	46.7	14.94	43.90 ± 11.92	67 ± 3.29
Vitamin D levels before supplementation (ng/ml) (Deficiency	11.4 (6.5-16.3)	10.9 (5.7-16.1)	10.77 ± 3.21	11.91 ± 3.88	-	-	11.73 ± 3.45	10.85 ± 3.31
Vitamin D levels before supplementation (ng/ml) (Insufficiency)	24.6 (20.9-28.3)	24.9 (21-28.8)	25.13 ± 3.45	27.56 ± 0.70	14.43	14.94	-	-

ACE-I, angiotensin-converting enzyme inhibitor; ARB, angiotensin receptor blockers; BMI, body mass index; COPD, chronic obstructive pulmonary disease; Vit D. vitamin D.

### Statistical analysis

RevMan(version 5.3; Copenhagen: The Nordic Cochrane Centre, The Cochrane Collaboration, 2014) was used for all statistical analyses. The results from trials were presented as risk ratios (RRs) to compare dichotomous outcomes while weighted mean difference (WMD) was calculated for continuous outcomes with 95% confidence intervals (CIs), and were pooled using a random effect model. Forest plots were created to assess visually the results of pooling. Outcomes of interest were incidence of postoperative atrial fibrillation and length of hospital stay in patients who underwent CABG. Heterogeneity across studies was evaluated using Higgins *I*^2^ and a value less than 50% for *I*^2^ was considered acceptable. The funnel plot was visually inspected to assess for publication bias. A *P-*value of less than 0.05 was considered significant in all cases.

## Results

### Literature search

**Figure 1.**
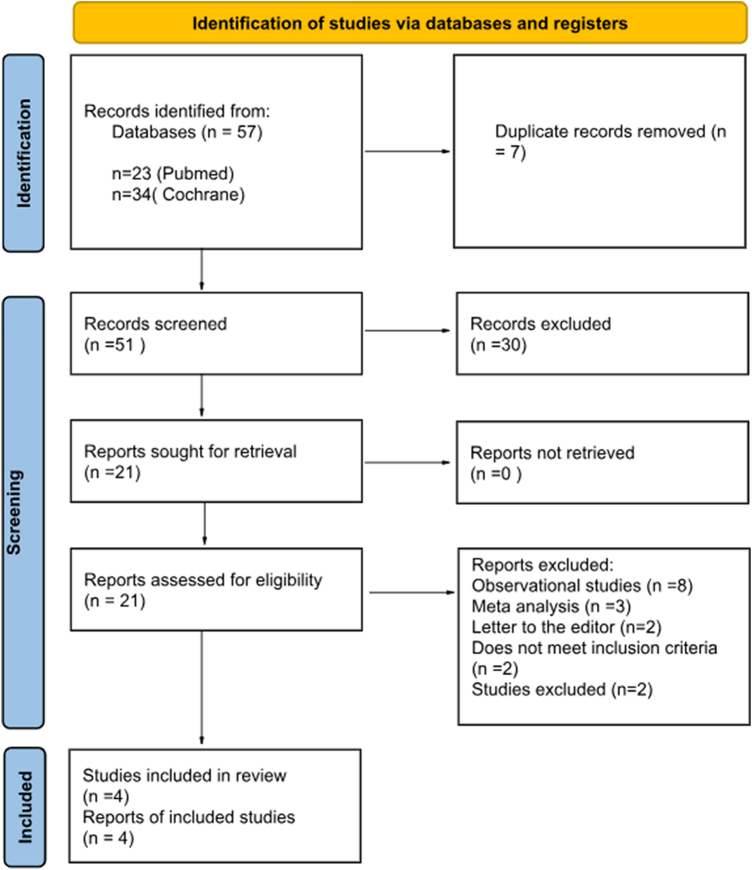
PRISMA flowchart outlining the literature search.

The initial search yielded a total of 57 articles. The final meta-analysis, after removing duplicates and screening for full text, included four RCTs Cerit, Kara, Talasaz, and Alirezaei^[17-20]^ involving 694 patients. All four studies showed very low risk of bias proving to be high quality studies (Supplementary Figure S1. http://links.lww.com/MS9/A838). The PRISMA flowchart outlines the screening process and exclusion criteria in detail (Fig. 1).

### Baseline characteristics

The mean age of the patients in the vitamin D group was 61.3 years while the control group was 62.2 years. In the intervention group, 3 days before surgery, patients received a daily dose of 150 000 units of vitamin D orally consisting of 50 000 units per tablet, three times a day, while the control group received placebo tablets (Alirezaaei *et al*)^[20]^. In another study, patients received a single oral dose of 50 000 IU of vitamin D (Cerit *et al*)^[17]^. In the study by Kara *et al*, patients with vitamin D deficiency received 300 000 IU of oral vitamin D, while those with insufficiency received 150 000 IU 48 hours before surgery^[18]^. In the study by Talasaz *et al*, patients received a single 600 000 IU dose due to the short time to surgery^[19]^. The mean hospital stay in vitamin D group was 10.58 days while in control group was 11.14 days. Mean Vitamin D levels in before supplementation (deficiency) in the intervention group were 11.3 while in the control group were 11.13. While the mean Vitamin D levels before supplementation (Insufficiency) in the intervention group were 21.38 while in the control group were 22.46. Baseline characteristics of the included studies are depicted in Table 1.

### Postoperative atrial fibrillation

**Figure 2.**
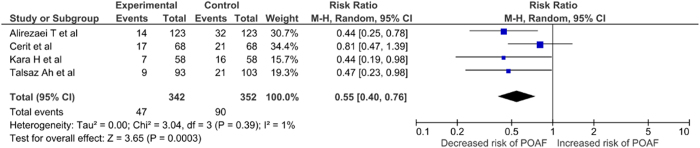
Forest plot displaying the incidence of atrial fibrillation after vitamin D in CABG patients.

Four studies compared the effectiveness of Vitamin D supplementation on postoperative atrial fibrillation. Pooling the studies shows that the Vitamin D supplementation significantly reduces the risk POAF (RR: 0.55 [0.40 to 0.76]; *P* = 0.0003; *I*^2^ = 1%) (Fig. 2).

### Length of hospital stay

**Figure 3.**
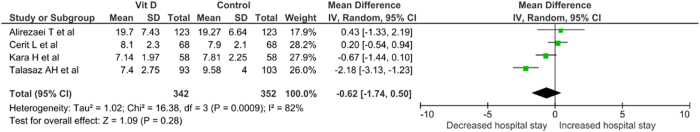
Forest plot displaying the length of hospital stay after vitamin D supplementation in CABG patients.

This forest plot suggests that there is no clear evidence that Vitamin D significantly affects hospital stay duration, (WMD: −0.62[−1.74, 0.50]; *P* = 0.28; *I*^2^ = 82%). High heterogeneity implies that the results varied significantly across the studies as depicted by Fig. 3. Sensitivity analysis showed that the overall effect of Vitamin D on hospital stay duration remains non-significant even after excluding Talasaz AH *et al*^[19]^. The combined mean difference is slightly closer to zero, and the heterogeneity is significantly reduced, suggesting that the excluded study contributed to the variability in the original analysis. However, the results still indicate no clear evidence that vitamin D significantly affects hospital stay duration (WMD: −0.14[−0.82, 0.53]; *P* = 0.22; *I*^2^ = 34%) shown in Fig. 3.

## Discussion

This meta-analysis of 694 patients undergoing CABG; with either vitamin D deficiency or insufficiency shows that vitamin D supplementation preoperatively was associated with a lower incidence of postoperative atrial fibrillation. No effect on length of hospital stay was noted

Our meta-analysis confirms that preoperative vitamin D supplementation is associated with a significant reduction in POAF incidence. This finding is consistent with earlier studies but is bolstered by the inclusion of additional participants, enhancing the robustness of the evidence. The presence of vitamin D receptors in cardiac muscle cells and its anti-inflammatory effects, such as reducing cardiac fibrosis and inflammation, likely contribute to this protective effect^[21,22]^. This result underscores the potential of vitamin D as a preventive measure in high-risk surgical populations, particularly those undergoing CABG. Our findings also align with studies reporting that correcting vitamin D deficiency through supplementation significantly reduces atrial electromechanical delay (AEMD), a known risk factor for AF^[23]^. The reduction in AEMD may contribute to the decreased risk of developing AF, reinforcing the role of vitamin D in maintaining normal cardiac rhythm^[23]^. This aspect of vitamin D’s action is particularly important, as it suggests a direct electrophysiological mechanism through which vitamin D exerts its protective effects against POAF. Vitamin D deficiency plays a significant role in modifying the heart’s electrophysiology, such as shortening the action potentials and the refractory period of the atrial myocardium through both direct and indirect actions. This results in elevated intracellular calcium levels within cardiac myocytes, which leads to an imbalance in electrolytes. This imbalance alters cardiac electrophysiology and contractility^[24,25]^. The study by Hanafy *et al* demonstrated an increase in action potential duration and contractility in the atrium following vitamin D administration, suggesting that it may be an effective therapy for preventing POAF^[26]^.

Contrary to the reduction in POAF, our analysis did not find clear evidence that vitamin D supplementation significantly affects hospital stay duration. The high heterogeneity observed in this outcome suggests variability across studies, which may stem from differences in study design, participant characteristics, or intervention protocols. A sensitivity analysis excluding the study by Talasaz AH *et al* reduced heterogeneity but did not alter the non-significant finding^[19]^. This highlights the complexity of assessing hospital stay duration as an outcome and suggests that while vitamin D may reduce POAF, its effect on hospitalization length is less clear and warrants further investigation.

Previous meta-analyses, including those by Hameed, Rahimi, and Liu, have explored the association between vitamin D and POAF, but the findings have been inconsistent due to variability in study designs, participant characteristics, and the timing of vitamin D measurements. Hameed’s analysis included three randomized controlled trials (RCTs) with 448 participants, suggesting a decrease in POAF and hospital stay with preoperative vitamin D supplementation. However, the small sample size limited the generalizability of these findings^[13]^. A previous meta-analysis by Hameed *et al* (2023) included three RCTs (*n* = 448) and reported a significant reduction in POAF incidence with vitamin D supplementation, along with a non-significant trend toward reduced hospital stay. Our study expands on these findings by incorporating an additional RCT, increasing the total sample size to 694. While our results similarly indicate a significant reduction in POAF, the reduction in length of hospital stay remained non-significant. Notably, our analysis exhibited greater heterogeneity in the LOS outcome, which may be attributed to differences in patient populations, vitamin D dosing regimens, timing of administration, and perioperative care protocols across the included studies. This highlights the need for further standardized research in this area. These contributions improve the understanding of vitamin D’s role in cardiac surgery and support its potential use in perioperative management strategies. Rahimi’s meta-analysis of five observational studies highlighted age as a significant confounding factor, emphasizing the need for careful matching in studies^[27]^. Liu’s larger meta-analysis with 74 885 participants identified Vitamin D deficiency as a moderate predictor of AF, yet heterogeneity remained a significant concern^[28]^. Our updated meta-analysis builds on this existing literature by incorporating a new RCT by Alirazei, increasing the total number of participants to 694^[20]^. This expansion strengthens the evidence base and addresses the gaps left by prior studies, particularly in the context of preoperative Vitamin D supplementation in CABG patients.

The findings of our meta-analysis suggest that preoperative Vitamin D supplementation could be a valuable strategy in reducing the incidence of POAF in patients undergoing CABG. This has significant implications for clinical practice, as it offers a relatively simple and low-cost intervention to mitigate a common and serious postoperative complication. However, our results also highlight the need for large-scale RCTs to confirm these benefits, determine the optimal dosage, and evaluate the long-term effects of vitamin D supplementation on POAF recurrence, cardiovascular morbidity, and mortality.

### Study limitations

Despite the strengths of our meta-analysis including greater sample size than the previous meta-analysis and all studies being randomised control trials, several limitations should be acknowledged. The high heterogeneity observed in the analysis of hospital stay duration suggests that factors such as study design and participant variability may have influenced the outcomes. Additionally, while our study expands the evidence base, the sample size remains limited due to the smaller number of included studies (*n* = 4), and the generalizability of our findings to other surgical populations or non-CABG patients is uncertain. Our study focused solely on CABG patients, and we did not assess the effect of vitamin D on other types of surgery. Moreover, there was no predetermined target vitamin D dose to reduce the incidence of atrial fibrillation, and the study was not pre-examined for underlying electrolyte abnormalities, which are one of the precipitating factors for atrial fibrillation. Lastly, our study does not specify the methods used to evaluate the incidence of atrial fibrillation. Clinical outcomes such as myocardial infarction (MI), incidence of survival, cardiac tamponade and cardiac arrest could not be assessed due to limited sample size. Additionally, while our study incorporated more recent evidence than prior meta-analyses, variability in study designs and outcome definitions may have contributed to increased heterogeneity, particularly regarding length of hospital stay. Future research should address these limitations by conducting well-designed, large-scale RCTs with standardized protocols to provide more definitive conclusions.

## Conclusion

Our updated pooled analysis concludes that preoperative vitamin D supplementation reduces the incidence of POAF in patients who underwent CABG. POAF occurs in approximately a quarter of CABG patients and leads to significant adverse outcomes and increased healthcare costs. Therefore, implementing effective preventive strategies could have substantial clinical and economic benefits, including integrating vitamin D in the preoperative regimen. Our analysis also shows that vitamin D supplementation does not significantly reduce the length of hospital stay. Therefore, its benefits could be specific and limited to arrhythmia prevention. Future studies, particularly large-scale randomized controlled trials with diverse patient populations, exploring a broader range of cardiovascular and non-cardiovascular outcomes are mandated to better understand the full impact of vitamin D supplementation in CABG patients and to refine its role in clinical practice.”

## Data Availability

Not applicable.

## Research registration unique identifying number (UIN)

NA National; Institute for Health Research (NIHR) International prospective register of systematic reviews (PROSPERO) (Identification No. CRD42024582058).

## References

[1] Dobrev D Aguilar M Heijman J. Postoperative atrial fibrillation: mechanisms, manifestations and management. Nat Rev Cardiol 2019;16:417–36. 30792496 10.1038/s41569-019-0166-5

[2] Ahlsson A Fengsrud E Bodin L. Postoperative atrial fibrillation in patients undergoing aortocoronary bypass surgery carries an eightfold risk of future atrial fibrillation and a doubled cardiovascular mortality. Eur J Cardiothorac Surg 2010;37:1353–59. 20138531 10.1016/j.ejcts.2009.12.033

[3] Potdar SP Shales S Baviskar M. Incidence, predictors, and outcome for post-operative atrial fibrillation in Indian patients undergoing off-pump coronary artery bypass grafting-a prospective observational study. Indian J Thorac Cardiovasc Surg 2022;38:366–74. 35756560 10.1007/s12055-022-01358-7 PMC9218032

[4] Lin MH Kamel H Singer DE. Perioperative/postoperative atrial fibrillation and risk of subsequent stroke and/or mortality. Stroke 2019;50:1364–71. 31043148 10.1161/STROKEAHA.118.023921

[5] Maesen B Nijs J Maessen J. Post-operative atrial fibrillation: a maze of mechanisms. Europace 2012;14:159–74. 21821851 10.1093/europace/eur208 PMC3262403

[6] Wang TJ Pencina MJ Booth SL. Vitamin D deficiency and risk of cardiovascular disease. Circulation 2008;117:503–11. 18180395 10.1161/CIRCULATIONAHA.107.706127 PMC2726624

[7] Schleithoff SS Zittermann A Tenderich G. Vitamin D supplementation improves cytokine profiles in patients with congestive heart failure: a double-blind, randomized, placebo-controlled trial. Am J Clin Nutr 2006;83:754–59. 16600924 10.1093/ajcn/83.4.754

[8] Emren SV Aldemir M Ada F. Does deficiency of Vitamin D increase new onset atrial fibrillation after coronary artery bypass grafting surgery? Heart Surg Forum 2016;19:E180–184. 27585197 10.1532/hsf.1531

[9] Özsin KK Sanrı US Toktaş F. Effect of plasma level of Vitamin D on postoperative atrial fibrillation in patients undergoing isolated coronary artery bypass grafting. Braz J Cardiovasc Surg 2018;33:217–23. 30043913 10.21470/1678-9741-2017-0214 PMC6089122

[10] Daie M Hajhossein Talasaz A Karimi A. Relationship between Vitamin D levels and the incidence of post coronary artery bypass graft surgery atrial fibrillation. J Tehran Heart Cent 2018;13:159–65. 30972113 PMC6450811

[11] Ohlrogge AH Brederecke J Ojeda FM. The relationship between vitamin d and postoperative atrial fibrillation: a prospective cohort study. Front Nutr 2022;9:851005. 35619954 10.3389/fnut.2022.851005 PMC9127673

[12] Hameed I Malik S Nusrat K. Effect of vitamin D on postoperative atrial fibrillation in patients who underwent coronary artery bypass grafting: a systematic review and meta-analysis. J Cardiol 2023;82:220–24. 37236436 10.1016/j.jjcc.2023.05.007

[13] Hutton B Salanti G Caldwell DM. The PRISMA extension statement for reporting of systematic reviews incorporating network meta-analyses of health care interventions: checklist and explanations. Ann Intern Med 2015;162:777–84. 26030634 10.7326/M14-2385

[14] Shea BJ Reeves BC Wells G. AMSTAR 2: a critical appraisal tool for systematic reviews that include randomised or non-randomised studies of healthcare interventions. Or Both BMJ 2017;358:j4008. 28935701 10.1136/bmj.j4008 PMC5833365

[15] International Prospective Register of Systematic Reviews (PROSPERO), National Institute for Health Research (NIHR), 2022. Accessed 25 August 2024. https://www.crd.york.ac.uk/PROSPERO/display_record.php?RecordID=341490.

[16] Higgins JPT Altman DG Gøtzsche PC. The Cochrane Collaboration’s tool for assessing risk of bias in randomised trials. BMJ 2011;343:d5928. 22008217 10.1136/bmj.d5928 PMC3196245

[17] Cerit L Özcem B Cerit Z. Preventive effect of preoperative Vitamin D supplementation on postoperative atrial fibrillation. Braz J Cardiovasc Surg 2018;33:347–52. 30184031 10.21470/1678-9741-2018-0014 PMC6122752

[18] Kara H Yasim A. Effects of high-dose vitamin D supplementation on the occurrence of post-operative atrial fibrillation after coronary artery bypass grafting: randomized controlled trial. Gen Thorac Cardiovasc Surg 2020;68:477–84. 31559589 10.1007/s11748-019-01209-0

[19] Talasaz AH Salehiomran A Heidary Z. The effects of vitamin D supplementation on postoperative atrial fibrillation after coronary artery bypass grafting in patients with vitamin D deficiency. J Card Surg 2022;37:2219–24. 35470909 10.1111/jocs.16550

[20] Alirezaei T Ansari Aval Z Karamian A. Effect of preoperative vitamin D on postoperative atrial fibrillation incidence after coronary artery bypass grafting. Gen Thorac Cardiovasc Surg 2024;72:649–55. 38485852 10.1007/s11748-024-02020-2

[21] de la Guía-galipienso F Martínez-Ferran M Vallecillo N. Vitamin D and cardiovascular health. Clin Nutr 2021;40:2946–57. 33397599 10.1016/j.clnu.2020.12.025 PMC7770490

[22] Graczyk S Grzeczka A Pasławska U. The possible influence of Vitamin D levels on the development of atrial fibrillation-an update. Nutrients 2023;15:2725. 37375629 10.3390/nu15122725 PMC10302036

[23] Canpolat U Yayla Ç Akboğa MK. Effect of Vitamin D replacement on atrial electromechanical delay in subjects with vitamin D deficiency. J Cardiovasc Electrophysiol 2015;26:649–55. 25772677 10.1111/jce.12656

[24] Zittermann A Kuhn J Ernst JB. 25-hydroxyvitamin D, 1,25-dihydroxyvitamin D and postoperative outcome in cardiac surgery. J Clin Endocrinol Metab 2015;100:72–80. 25365313 10.1210/jc.2014-3013

[25] Alonso A Misialek JR Michos ED. Serum 25-hydroxyvitamin D and the incidence of atrial fibrillation: the atherosclerosis risk in communities (ARIC) study. Europace 2016;18:1143–49. 26847078 10.1093/europace/euv395 PMC4974632

[26] Hanafy DA Chang SL Lu YY. Electromechanical effects of 1,25-dihydroxyvitamin d with antiatrial fibrillation activities. J Cardiovasc Electrophysiol 2014;25:317–23. 24152033 10.1111/jce.12309

[27] Rahimi M Taban-Sadeghi M Nikniaz L. The relationship between preoperative serum vitamin D deficiency and postoperative atrial fibrillation: a systematic review and meta-analysis. J Cardiovasc Thorac Res. 2021;13:102–08. 34326963 10.34172/jcvtr.2021.25 PMC8302893

[28] Liu X Wang W Tan Z. The relationship between vitamin D and risk of atrial fibrillation: a dose-response analysis of observational studies. Nutr J. 2019;18:73. 31727055 10.1186/s12937-019-0485-8 PMC6857145

